# The use of nerve and muscle biopsy in the diagnosis of vasculitis: a 5 year retrospective study

**DOI:** 10.1136/jnnp.2008.151126

**Published:** 2008-09-26

**Authors:** D L H Bennett, M Groves, J Blake, J L Holton, R H M King, R W Orrell, L Ginsberg, M M Reilly

**Affiliations:** 1 Wellcome Clinical Scientist Fellow King’s College and University College London, London, UK; 2 MRC Centre for Neuromuscular Diseases, Department of Molecular Neurosciences, National Hospital for Neurology and Neurosurgery and Institute of Neurology, London, UK; 3 Department of Clinical Neurosciences, Institute of Neurology, Hampstead Campus, University College London and Royal Free Hospital, London, UK; 4 Department of Neurophysiology, Norfolk and Norwich University Hospital, Norwich, UK

## Abstract

**Introduction:**

Peripheral nerve vasculitis is an important condition which can be diagnostically challenging and is one of the principal current indications for nerve and muscle biopsy. Previous studies have suggested that combined nerve and muscle biopsy (usually of the superficial peroneal nerve and peroneus brevis muscle) produces a higher diagnostic yield than nerve biopsy alone in the investigation of vasculitis.

**Objective:**

To determine whether in our two centres combined nerve (usually the sural) and muscle (usually the vastus lateralis) biopsy improved diagnostic yield compared with nerve biopsy alone.

**Methods:**

We interrogated our database of all nerve biopsies (usually of the sural nerve) performed at our institutions over 5 years and identified 53 cases of biopsy proven peripheral nerve vasculitis. Clinicopathological and neurophysiological data in these patients were reviewed.

**Results:**

The most common clinical presentation was with a painful asymmetric axonal polyneuropathy or mononeuritis multiplex (66% of cases). Nerve biopsy demonstrated definite vasculitis in 36%, probable vasculitis in 62% and no vasculitis in 2% of cases. In 24 patients a muscle biopsy (usually the vastus lateralis) was also performed and vasculitis was demonstrated in 46% of these (in 13% showing definite and 33% probable vasculitis). There was only one patient in whom vasculitis was demonstrated in muscle but not in peripheral nerve.

**Conclusion:**

Combined nerve (usually sural) and vastus lateralis muscle biopsy did not significantly increase the diagnostic yield compared with nerve biopsy alone. A sensible approach to the diagnosis of peripheral nerve vasculitis is to choose a nerve to biopsy which is clinically affected and amenable to biopsy. If the sural nerve is chosen, the data suggest that it is not routinely worth doing a vastus lateralis biopsy at the same time, whereas if the superficial peroneal nerve is chosen, it seems appropriate to do a combined superficial peroneal nerve and peroneus brevis biopsy. It is still not known if both the sural and superficial peroneal nerves are involved clinically which one gives the higher yield if biopsied.

Peripheral nerve vasculitis is a pathological process involving infiltration of and injury to the walls of the vasa nervorum by inflammatory cells,^1^ ^2^ resulting in secondary ischaemia and damage to the nerve trunk. The diagnosis is difficult to make on clinical grounds alone and usually requires the biopsy of affected tissue prior to starting treatment. Although peripheral nerve vasculitis is rare, it remains one of the most important indications for performing a nerve biopsy. Peripheral nerve vasculitis can occur either as part of multisystem disorders such as polyarteritis nodosa or connective tissue disorders (termed SVN, systemic vasculitic neuropathy)^1^ ^3^^–^5 or as a disorder restricted to the peripheral nervous system (termed NSVN, non-systemic vasculitic neuropathy).^6^^–^8 In both groups, striated muscle as well as peripheral nerve may show pathological signs of vasculitis. In 1988, Said and Lacroix reported that up to 45% of patients with peripheral nerve vasculitis had demonstrable evidence of vasculitis in the peroneus brevis muscle (PBM) but not in the superficial peroneal nerve (SPN) when both were biopsied.^4^ This group therefore suggested that combined SPN/PBM biopsy (through a common incision) in patients with suspected peripheral nerve vasculitis would increase the diagnostic yield, an approach supported by more recent studies.^1^ ^9^ Based on these studies it has also become accepted practice in units where the sural nerve is the more usual nerve biopsied to biopsy the vastus lateralis muscle as well (using a second incision during the same procedure) when vasculitis is being considered.

In order to determine whether combined nerve (usually sural) and vastus lateralis muscle biopsy improved diagnostic yield compared with nerve biopsy alone, we studied more than 50 cases of pathologically confirmed peripheral nerve vasculitis seen at our two institutions over a 5 year period.

## METHODS

### Case identification

We interrogated our databases of all nerve and muscle biopsies performed at the National Hospital for Neurology and Neurosurgery, Queen Square, London, and the Royal Free Hospital, London, between January 1999 and August 2005, in order to identify all cases of pathologically confirmed vasculitis.

### Tissue processing

Nerve biopsies were of the superficial peroneal, superficial radial or sural nerve; muscle biopsies were taken from either the vastus lateralis or peroneus brevis muscles. Following biopsy, part of the nerve was frozen in liquid nitrogen or processed into paraffin wax, and another piece embedded in epoxy resin using standard protocols. Teased nerve fibre preparations were performed in selected patients. Paraffin sections were stained with haematoxylin–eosin and elastic Van Gieson, as well as a panel of antibodies, including anti-CD45RO and anti-CD3 (for T lymphocytes) and anti-CD68 for macrophages. Frozen sections were stained immunocytochemically for B lymphocytes (CD22), T lymphocytes (CD4 and CD8), early macrophage marker (CD68) and late macrophage product (Mac387). Epoxy resin sections were stained with methylene blue–azure A or thionine and acridine orange, and areas selected for electron microscopy sectioned at 70 nm and stained with uranyl acetate and lead citrate.

Muscle biopsies were frozen in isopentane cooled with liquid nitrogen. Cryosections (10 μm) were cut, mounted on coverslips and dried at room temperature for 1 h. Sections were stained with haematoxylin–eosin and Gomori trichrome, as well as with a routine panel of histochemical stains. Immunohistochemical staining for CD3, CD8, CD4, CD20 and CD68 was performed using standard methods.

### Pathological selection criteria

Nerve biopsies were classified as showing definite or probable vasculitis. Definite vasculitis was diagnosed if endoneurial or epineurial vessels showed evidence of vessel wall infarction in association with perivascular or transmural infiltration by inflammatory cells. Vessel wall infarction was diagnosed if there was evidence of destruction and disorganisation of the muscularis by fibrinoid necrosis, disruption of the endothelium or internal elastic lamina, thrombosis of the lumen or haemorrhage into the wall of the vessel. Probable vasculitis was diagnosed if there was transmural or perivascular inflammation not accompanied by vessel wall infarction but associated with at least one of the following: fibrous scarring/intimal proliferation, chronic organised thrombosis (with/without recanalisation), haemosiderin deposits, prominent Wallerian degeneration or asymmetric nerve fibre loss. In muscle biopsy specimens, similar diagnostic criteria for definite vasculitis were applied. Probable vasculitis was diagnosed where transmural inflammation was not accompanied by fibrinoid necrosis of the vessel wall or any of the other vascular changes described above as representing evidence of definite vasculitis. Note that in muscle, transmural inflammation alone without the additional features which were applied to nerve biopsies was sufficient to diagnose probable vasculitis. This was because some of these features (prominent Wallerian degeneration or asymmetric nerve fibre loss) can only be applied to nerve and we felt such a definition would be overly restrictive when applied to muscle.

### Clinical and electrophysiological data

Clinical case notes were reviewed to provide clinical and electrophysiological data for all patients meeting the pathological criteria for vasculitis. All patients were clinically suspected of having vasculitis. The pattern of neuropathy was determined by the findings on clinical examination. Nerve conduction studies and electromyography were performed routinely prior to biopsy, using standard techniques. Neurophysiological examination was performed in more than one laboratory and by a number of different neurophysiologists. If the sural nerve sensory action potential (SAP) was either reduced or absent, this nerve was biopsied; if not, an alternative affected nerve was chosen. The one exception to this was a patient with a small fibre neuropathy with normal neurophysiology and in whom the sural nerve territory was clinically affected (and therefore biopsied). In all cases, the biopsied nerve was clinically affected. All muscle biopsies were taken from the vastus lateralis, apart from one taken from the PBM.

Patients with pathologically confirmed vasculitis were divided into SVN and NSVN groups. The criteria that were used to define cases as NSVN were as follows: (1) no evidence of involvement outside the peripheral nervous system (except striated muscle) and (2) no underlying causative agent (hepatitis B, hepatitis C, HIV, drug exposure, connective tissue disorder, malignancy or cryoglobulinaemia). Patients with diabetes mellitus were not excluded. Systemic vasculitis was defined as in the Chapel Hill Consensus Conference.^10^ Constitutional symptoms such as fever and weight loss and serological tests such as antinuclear antibodies (ANA), antineutrophil cytoplasmic antibodies (ANCA), erythrocyte sedimentation rate (ESR) and rheumatoid factor (RF) were not in themselves used to diagnose SVN unless independent clinical criteria for the diagnosis of a connective tissue disorder were present.

Statistics were performed using SigmaStat 3.5 software. Categorical variables were analysed using the χ^2^, Fisher’s exact test or McNemar’s test where appropriate. The Mann–Whitney rank sum test was used to compare the duration of symptoms prior to biopsy in SVN and NSVN cohorts as the data were not normally distributed.

## RESULTS

### Clinical features

Fifty-three patients were identified on the basis of a pathologically confirmed diagnosis of vasculitis between January 1999 and August 2005. The clinical characteristics of our cohort of patients are summarised in table 1 and a breakdown of all patients is shown in supplementary table 1 (available online). Thirty-one patients (58%) had SVN and 22 (42%) NSVN. There was a female preponderance (women:men 30:23). Age range at the time of biopsy was 32–79 years (mean 56 (SD 13)). Duration of symptoms prior to biopsy ranged from 1 to 144 months (mean 15 (SD 23), median 6). Mean duration of symptoms prior to biopsy was shorter in the SVN than in the NSVN group (8.5 vs 23.5 months, respectively) but this difference was not statistically significant (Mann–Whitney rank sum test, p = 0.36). The majority (87%) of neuropathies were painful on presentation and the most common presentation was with either an asymmetric sensorimotor neuropathy or mononeuritis multiplex (45% and 20% of patients, respectively). Nine patients (17%) had only sensory findings on presentation (one with a pure small fibre neuropathy). One patient presented with a bilateral asymmetric brachial and another with bilateral asymmetric lumbosacral plexopathy. Only four patients (8%) had cranial nerve involvement (third cranial nerve in one patient, fifth cranial nerve in two patients, and sixth cranial nerve in one patient). Table 2 shows the frequency of involvement of different motor nerves, as determined by clinical examination. Supplemental table 2 (available online) shows the same data divided into SVN versus NSVN groups. Note that for the purposes of table 2, the observed deficits were decomposed into constituent peripheral nerves (sciatic nerve involvement in two patients was reduced to peroneal and tibial). There was a distal predominance. The most commonly involved nerve was the peroneal (86% of patients); in the upper limbs the most commonly involved nerve was the ulnar (63% of patients).

**Table 1:** Characteristics of the vasculitic neuropathy cohort analysed collectively and by vasculitis group

	All patients (n = 53)	SVN (n = 31)	NSVN (n = 22)
Clinical			
Age (year)*	56 (12.6)	55 (12.4)	59 (12.7)
Sex (Men:Women)	23:30	14:17	9:13
Duration (months)* (median)	14.8 (23.3) (6)	8.5 (8.3) (6)	23.5 (33) (8)
Systemic Sx	33 (62)	28 (90)	5 (23)
Pain	46 (87)	27 (87)	19 (86)
Clinical course P:SW	19:34	9:22	10:12
Neuropathy pattern			
ASYSM	24 (45)	15 (48)	9 (41)
ASYS	7 (13)	3 (10)	4 (18)
MM	11 (20)	8 (26)	3 (14)
SYSM	6 (11)	2 (6)	4 (18)
Other	bLSP 1, bBRP 1, M 1, SFN 1, SYS 1	bLSP 1, M 1, SYS 1	bBRP 1, SFN 1
CN	4 (8)	2 (6)	2 (9)
Laboratory data			
Anaemia	13/53 (25)	12/31 (39)	1/22 (5)
ESR (mm/h)*	39 (36)	52 (36)	23 (27)
ESR >20 mm/h	26/49 (53)	20/28 (71)	6/21 (29)
ANA pos	7/52 (13)	6/30 (20)	1/22 (5)
ANCA pos	10/52 (19)	10/31 (32)	0/21
RF positive	12/50 (24)	10/29 (28)	2/21 (9)
↑CSF protein	8/23 (35)	7/13 (54)	1/10 (10)
↑CSF white blood cell count	4/23 (17)	3/13 (23)	1/10 (10)
OCB +ve	6/22 (27)	5/12 (42)	1/10 (10)
Nerve pathology			
Definite vasculitis	19/53 (36)	15/31 (48)	4/22 (18)
Probable vasculitis	33/53 (62)	16/31 (52)	17/22 (77)
No vasculitis	1/53 (2)	0/31 (0)	1/22 (5)
Wallerian degeneration	45/53 (85)	26/31 (84)	19/22 (86)
Asymmetric nerve fibre loss	17/53 (32)	8/31 (26)	9/22 (41)
Haemosiderin	11/53 (21)	8/31 (26)	3/22 (14)
Fibrous obliteration±recanalisation	15/53 (28)	11/31 (35)	4/22 (18)
Muscle pathology			
Definite vasculitis	3/24 (13)	3/17 (18)	0/7 (0)
Probable vasculitis	8/24 (33)	6/17 (35)	2/7 (29)
No vasculitis	13/24 (54)	8/17 (47)	5/7 (71)
Inflam infiltrate but no frank vasculitis	5/24 (21)	2/17 (12)	3/7 (43)
Myofibre necrosis	5/24 (21)	3/17 (18)	2/7 (29)
Neurogenic change	15/24 (63)	12/17 (71)	3/7 (43)

Data expressed as *mean (SD) or n (%) unless otherwise indicated.

ANA, antinuclear antibodies; ANCA, antineutrophil cytoplasmic antibodies; ASYS, asymmetric sensory neuropathy; ASYSM, asymmetric sensorimotor neuropathy; bBRP, bilateral brachial plexopathy; bLSP, bilateral lumbosacral plexopathy; CN, cranial nerve involvement; Duration, duration of symptoms prior to biopsy; ESR, erythrocyte sedimentation rate; M, mononeuropathy; MM, mononeuritis multiplex; NSVN, non-systemic vasculitic neuropathy; OCB+, unmatched oligoclonal bands in cerebrospinal fluid; P, progressive; RF, rheumatoid factor; SFN, small fibre neuropathy; SVN, systemic vasculitic neuropathy; SW, stepwise; Sx, symptoms; SYS, symmetrical sensory neuropathy; SYSM, symmetrical sensorimotor neuropathy.

**Table 2:** Frequency of motor nerve involvement in those patients with vasculitic neuropathy who demonstrated a motor deficit (patients with pure sensory neuropathy were excluded from the denominator)

Nerve	% per patient	% per nerve
Musculocutaneous	9	9
Femoral	32	26
Radial	35	30
Tibial	51	40
Median	58	52
Ulnar	63	55
Peroneal	86	76

In the NSVN group, 16% of patients suffered weight loss and 5% had fever. As expected, systemic features were much commoner in the SVN group (p<0.001, comparing the proportion of patients with systemic symptoms SVN versus NSVN; Fisher’s exact test), in which weight loss occurred in 52% and fever in 18%. In the SVN group there were a number of additional features, including: arthralgia (26%), rash (23%), renal (19%), respiratory (19%) and gastrointestinal involvement (16%). The diagnoses of those patients suffering from SVN included microscopic polyangiitis (n = 4 patients), Churg–Strauss syndrome (n = 3), hepatitis B (n = 3), polyarteritis nodosa (n = 3), rheumatoid vasculitis (n = 3), Wegener’s granulomatosis (n = 3), hepatitis C (n = 2), HIV (n = 2), paraneoplastic secondary to small cell lung cancer (n = 2), Sjögren’s syndrome (n = 2), undifferentiated connective tissue disorder (n = 2), chronic graft versus host disease following bone marrow allograft for non-Hodgkin’s lymphoma (n = 1) and systemic sclerosis (n = 1).

### Laboratory findings

Laboratory findings are summarised in table 1. As expected, patients with SVN compared with those with NSVN were significantly more likely to be anaemic (p = 0.004, Fisher’s exact test), to have a raised ESR (p = 0.01, χ^2^ test) and to have positive serology for ANCA (p = 0.003, Fisher’s exact test) (see table 1). A greater proportion of patients with SVN had positive ANA and RF serology compared with patients with NSVN but these differences did not reach significance. In one of the patients with paraneoplastic neuropathy secondary to small cell lung carcinoma, antineuronal antibodies were tested and found to be negative. Twenty-three of our total vasculitis cohort had a lumbar puncture, of which 11 were abnormal (eight had a CSF protein concentration >0.65 g/dl, six had unmatched oligoclonal bands and four had a raised CSF white cell count).

### Neurophysiology

The neurophysiology is summarised in supplementary table 3 (available online). For each individual study, a brief description of motor and sensory conduction as well as needle electromyography is given and the main clinical conclusion of the examining neurophysiologist is summarised. The most frequent finding was that of an asymmetric or patchy axonal motor and sensory peripheral neuropathy (23 patients, 43%). Relatively infrequent mononeuropathies were identified (four patients, 8%). Some patients were reported to have had a symmetrical or generalised neuropathy (13 patients, 25%). Occasional borderline motor slowing was identified (four patients had mixed features) but rarely in the frankly demyelinating range (less than 38 m/s in the upper limbs). In all cases of marked motor slowing there was evidence of significant or severe loss of motor axons. There were no cases with convincing findings of demyelination and no cases of partial motor conduction block. Nine patients (17%) had a principally sensory neuropathy.

### Pathological findings

**Figure 1:**
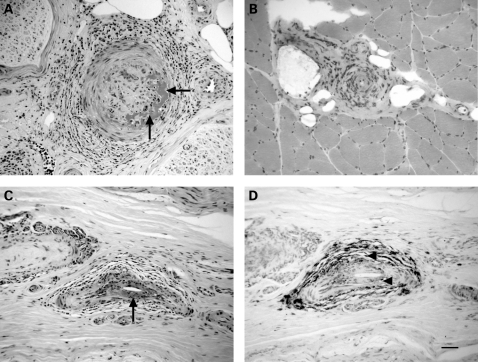
(A) 4 μm paraffin sections of sural nerve and (B) 10 μm frozen sections of muscle from case No 17 and (C, D) sural nerve from case No 11, stained with haematoxylin and eosin (A, B, C) or immunostained for CD45RO (D). In (A) the epineurial arteriole is occluded, and in (A) and (C), regions of fibrin deposition are present in the vessel wall (arrows). In (A), (B) and (C), mononuclear inflammatory cells surround the blood vessel, and immunohistochemistry for the pan T cell markers CD45RO and CD3 showed that they were also present within the vessel wall (D, arrowheads). Scale bar = 50 μm

Figure 1 shows selected examples of vasculitis within nerve and muscle biopsy specimens. From 53 patients there were 50 sural, three superficial radial and one superficial peroneal nerve biopsy (note that one patient had two nerve biopsies—a sural followed by a superficial radial). In 19 patients (36%) the nerve biopsy demonstrated definite and in 33 patients (62%) probable vasculitis. In patients with SVN the nerve biopsy was more likely to show definite (as opposed to probable) vasculitis when compared with NSVN patients (48% vs 18%, p = 0.04, Fisher’s exact test). Twenty-four patients had a muscle biopsy at the same time as their nerve biopsy (23 from the vastus lateralis and one from the PBM). The muscle biopsy demonstrated vasculitis in 11 cases (46% of all muscle biopsies and 48% of the vastus lateralis biopsies); three (13%) muscle biopsies showed definite and eight (33%) probable vasculitis. None of the muscle biopsies demonstrating probable vasculitis had accompanying signs of remote vascular injury (fibrous scarring/intimal proliferation, chronic organised thrombosis with or without recanalisation). Of those muscle biopsies which did not demonstrate vasculitis, five showed an inflammatory cell infiltrate. Five muscle biopsies (21%) showed myofibre necrosis and 15 (63%) showed neurogenic changes.

There was only one patient (with NSVN) in whom probable vasculitis was demonstrated in muscle but not in peripheral nerve. This patient had undergone a sural nerve biopsy 4 years prior to undergoing combined superficial radial nerve and vastus lateralis muscle biopsy (in both cases the biopsied nerve demonstrated evidence of clinical involvement and the relevant SAP was reduced or absent). Both nerve biopsies showed an inflammatory cell infiltrate, asymmetric nerve fibre loss and acute Wallerian degeneration. However, the inflammatory cell infiltrate was not centred on blood vessels and therefore did not meet our diagnostic criteria for definite or probable vasculitis. In the muscle biopsy there was a small focus of perivascular and intramural inflammation without evidence of fibrinoid necrosis, which was classified as probable vasculitis. Combined nerve and muscle biopsy did not significantly increase the proportion of patients diagnosed with vasculitis compared with nerve biopsy alone (p = 1.00, McNemar’s test). Two patients with SVN had skin biopsies which showed evidence of vasculitis, and one patient with Churg–Strauss syndrome had a laparoscopy and an omental biopsy showed vasculitis associated with granuloma formation.

## DISCUSSION

The demographic features of our vasculitis cohort were similar to previous studies, showing a female predominance and a tendency for the condition to affect the elderly.^5^ ^7^ ^11^^–^14 In agreement with previous reports,^3^ ^6^ ^7^ ^15^ ^16^ we have found that pain is a prominent symptom described by the vast majority (87%) of our patients. Most of our patients presented with an asymmetric neuropathy, usually either an asymmetric sensorimotor neuropathy (45%) or mononeuritis multiplex (20%). Only 11% presented with a symmetrical sensorimotor neuropathy. Reports of the proportion of patients presenting with symmetrical compared with asymmetric findings in the literature vary greatly from 2% of patients having a symmetrical neuropathy in one recent study of NSVN^6^ up to 76%^17^ in a study involving patients with SVN and NSVN. These discrepancies may relate to the extent to which minor asymmetries on examination are taken into account, or to the different patient populations. No patient in our cohort had a purely motor neuropathy although 15% presented with a pure sensory neuropathy, a proportion similar to previous reports.^6^ ^7^ ^9^ ^12^ ^18^ ^19^ Very few of our patients (8%) had cranial nerve involvement. The most commonly involved nerve in the lower limbs was the peroneal nerve and in the upper limbs the ulnar nerve. The frequent involvement of the peroneal nerve is compatible with experimental evidence demonstrating that the sciatic nerve bifurcation is a watershed zone, being particularly susceptible to ischaemia.^20^

Peripheral nerve vasculitis can occur either as part of a multisystem disorder (SVN) or as a disorder restricted to the peripheral nervous system (NSVN). In our cohort, patients with SVN had a shorter duration between symptom onset and nerve biopsy, more systemic symptoms and were more likely to have anaemia, raised inflammatory markers and positive serology for ANA, ANCA or RF than patients with NSVN.

It can be difficult to compare published cohorts of peripheral nerve vasculitis as different definitions of vasculitis have been used. In this study, we categorised peripheral nerve vasculitis into definite and probable. In definite vasculitis, there is evidence of both vascular inflammation as well as recent damage to the vessel wall. In probable vasculitis, there are transmural or perivascular inflammatory cells in combination with other features suggestive of vasculitis, such as prominent Wallerian degeneration, asymmetric nerve fibre loss or evidence of previous vascular injury (haemosiderin deposition or fibrous obliteration with or without recanalisation). A number of previous series have also subdivided cases into definite and probable vasculitis^6^ ^7^ while some have been more restrictive by including only those cases in which there is evidence of both vessel wall inflammation and necrosis.^4^ The inclusion of cases in which the nerve biopsy shows evidence of vessel wall inflammation without frank necrosis but with other features suspicious of vasculitis (ie, asymmetric nerve fibre loss, prominent Wallerian degeneration, predominant axonal changes) has been shown to increase the estimated sensitivity of the procedure from 61% to 86% with only a small loss of specificity.^12^

It was first reported in 1988 that combined nerve and muscle biopsy using superficial peroneal nerve (SPN) and peroneus brevis muscle (PBM) could increase the diagnostic yield compared with nerve biopsy alone.^4^ Of 83 patients who had the combined procedure, 37 (45%) had vasculitis in muscle but not in nerve and, overall, muscle biopsy was diagnostic for necrotising arteritis in 80% and nerve biopsy in 55% of cases. In a more recent review, the same authors described a larger cohort of 425 patients in which vasculitic lesions were found in muscle only in 28% of patients, nerve only in 45% and both in 31.5% of patients.^1^ A number of other groups^9^ ^12^ ^21^ describing combined SPN and PBM biopsy in the diagnosis of vasculitis have also found a sizeable percentage of patients in whom vasculitis is present in muscle but not nerve (varying between 9% and 27%). There are fewer evaluations of diagnostic yield when combining sural nerve biopsy with muscle biopsy. In 33 patients described as part of a cohort selected for the presence of muscle vasculitis (principally gastrocnemius), vasculitis was not found in the sural nerve in 20% of cases.^13^ Claussen *et al* described a series of 115 combined sural nerve and muscle biopsies (principally tibialis anterior and gastrocnemius) performed for suspected vasculitis. Histopathological evidence of vasculitis was found in 39% of cases and in agreement with our own findings, combined muscle biopsy did not improve diagnostic yield (there were no cases where vasculitis was demonstrated in muscle but not in nerve).^22^

Our policy is to use neurophysiology to target nerve biopsy and we preferentially chose the sural nerve. If the sural nerve is clinically involved and the SAP is reduced or absent, then this nerve is biopsied. If the sural nerve SAP is normal, an alternative affected nerve is used. Twenty-four patients had a simultaneous muscle biopsy (23 from the vastus lateralis and one from the PBM). In contrast with a number of previous studies, we only found a small increase in diagnostic yield when performing combined nerve and muscle biopsy. Only one patient (4% of all patients with the simultaneous procedure) had evidence of probable vasculitis present in muscle but not in nerve.

There are potentially a number of reasons why we found only a small increase in the diagnostic yield from combined nerve and muscle biopsies in our study. In the vast majority of cases we have biopsied a proximal muscle (vastus lateralis) while most other groups have biopsied more distal muscles, such as either the PBM or gastrocnemius. There could be a distal predominance for muscle vasculitis. In our series, 46% of the muscle biopsies from patients with peripheral nerve vasculitis showed vasculitis. This is much lower than the figure of 80% using PBM described by Said and colleagues,^4^ although two other groups have found results which vary between 31% and 59%.^9^ ^12^ A second possibility is the physical proximity of the SPN and PBM versus the remoteness of the sural nerve and vastus lateralis. It is possible that a contiguous muscle to an affected nerve is more likely to demonstrate vasculitis than a remote muscle, although this has not been studied. These differences may relate to the different nerves being biopsied. The sensitivity of SPN/PBM biopsy for vasculitis has been estimated at 60–70%^12^ ^18^ and the sensitivity of sural nerve biopsy is given as 50%.^7^ ^23^ However, it is difficult to draw conclusions given the different patient groups and definitions of vasculitis used in these studies. One study of NSVN patients did compare the sensitivity of SPN/PBM versus sural nerve biopsy in the diagnosis of definite vasculitis and found increased sensitivity of 58% versus 47%, respectively, but this was not statistically significant.^6^ A recent study comparing complications following SPN/PBM versus sural nerve biopsy has shown that although SPN biopsy can lead to a greater area of sensory loss compared with sural nerve biopsy, there is very little difference in other complications.^24^ Differences in the published diagnostic yield of combined nerve and muscle biopsy may also relate to the case mix (eg, the proportion of SVN versus NSVN cases) and the stringency of the criteria used to define vasculitis in peripheral nerve and muscle. The fact that in our two centres only 50% of patients who had a nerve biopsy for the investigation of vasculitis also had a combined muscle biopsy does introduce a source of potential bias into our study.

In conclusion, the practice of combined nerve and muscle biopsy when peripheral nerve vasculitis is suspected has arisen because of studies demonstrating the increased diagnostic yield when SPN and PBM are simultaneously biopsied. Our study clearly shows that the routine biopsy of the vastus lateralis simultaneously with a sural nerve biopsy does not significantly increase the diagnostic yield in cases of suspected peripheral nerve vasculitis. The crucial next question to be addressed is whether combined SPN/PBM is truly superior to a sural nerve biopsy alone when peripheral nerve vasculitis is suspected. The only way of definitively answering this question would be to perform a randomised prospective study in patients with suspected vasculitis who had evidence clinically and neurophysiologically of both sural nerve and peroneal nerve involvement. In addition, it would be important to compare the morbidity of both procedures. In the meantime, we suggest choosing a nerve to biopsy that is clinically and neurophysiologically affected. There is insufficient evidence at present to preferentially suggest either the sural nerve or combined SPN/PBM biopsy if both nerves are affected clinically. If the sural nerve is the nerve chosen to be biopsied to diagnose vasculitis, then it is not routinely worth doing a vastus lateralis biopsy at the same time. If the sural nerve biopsy does not demonstrate vasculitis but the clinical suspicion remains high, then another nerve and/or a muscle biopsy (eg, SPN/PBM) should be considered.
